# Detecting polynucleotide motifs: Pentads, hexads, and beyond

**DOI:** 10.1371/journal.pcbi.1013633

**Published:** 2025-10-28

**Authors:** Michal Zurkowski, Maja Marusic, Dorota Gudanis-Sobocinska, Marta Szachniuk

**Affiliations:** 1 Institute of Computing Science, Poznan University of Technology, Poznan, Poland; 2 Slovenian NMR Centre, National Institute of Chemistry, Ljubljana, Slovenia; 3 Institute of Bioorganic Chemistry, Polish Academy of Sciences, Poznan, Poland; University of Missouri, UNITED STATES OF AMERICA

## Abstract

The structural diversity of nucleic acids extends far beyond the canonical Watson–Crick base pairing, encompassing higher-order motifs, such as triads, tetrads, pentads, hexads, heptads, and octads, which play critical roles in genome regulation and stability. Among these, tetrads – forming the core of G-quadruplexes – and their polyadic extensions have emerged as key determinants in fundamental processes ranging from replication and transcription to telomere maintenance. However, the detection and characterization of these complex motifs in experimental structures remain challenging. To address this, we present LinkTetrado, a computational tool for the automated identification and classification of polyadic motifs in nucleic acid 3D structures. Applied to a curated dataset of 529 nucleic acid structures, LinkTetrado identified 25 unique structures containing such motifs, including previously unreported pentads, hexads, heptads, and octads in both DNA and RNA. Manual validation using NMR restraints and chemical shift data confirmed the accuracy of motif assignments and underscored the importance of integrating experimental evidence for reliable detection. LinkTetrado achieves a precision of 0.87 in identifying polyads. The tool is freely available at https://github.com/michal-zurkowski/linktetrado and provides a foundation for the systematic exploration of higher-order nucleic acid motifs.

## Introduction

**Fig 1:**
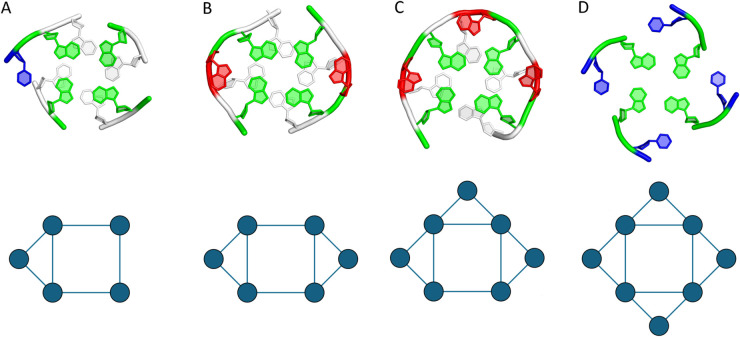
Example polyadic motifs of different orders and their schematic representations: (A) pentad (5 nucleotides), (B) hexad (6 nucleotides), (C) heptad (7 nucleotides), and (D) octad (8 nucleotides). In the 3D models (top row), nucleotides forming the polyads are shown in color, coded by nucleotide type: green for guanine, blue for uracil or thymine, and red for adenine. Green thus highlights the guanine tetrad core.

Nucleic acids, the fundamental carriers of genetic information, are renowned for their iconic double-helical structure stabilized by Watson-Crick-Franklin (WCF) base pairs. Yet both DNA and RNA can adopt a remarkable variety of alternative structures beyond this form, enabled by non-WCF base pairing and their expansion into higher-order, planar motifs called polyads or base-multiplets. Polyadic motifs – such as triads (3 bases), tetrads (4), pentads (5), hexads (6), heptads (7), and octads (8) – are formed when three or more nucleotides interact through hydrogen bonds, typically via their Hoogsteen or sugar edges, to create stable, ring-like networks where each base usually bonds with two neighbors [1]. Polyads significantly contribute to the stability and structural diversity of nucleic acids and are implicated in key processes, including gene regulation and molecular recognition. Fig 1 presents schematic models of example polyadic motifs analysed in this work.

Among polyads, tetrads have received the most attention due to their ability to stack into higher-order structures called quadruplexes. The most frequent and biologically relevant motif of this kind is the G-quadruplex (G4), formed by stacked G-tetrads. G4s are found in guanine-rich DNA and RNA sequences, notably within telomeres, promoter regions, and other functionally important genomic loci. They play critical roles in fundamental processes such as replication, transcription, translation, and telomere maintenance [2–6]. The biological outcome of G4 formation depends on factors including their topology, context, and the specific proteins that bind to them, with recognition influenced by both conserved elements and significant structural variability. Our understanding of G4s remains limited, as loops and flanking sequences, in addition to the G-quartet core, can dramatically affect their folding and final structure. Therefore, it is crucial to explore not only the central tetrads but also related features that modulate G4 properties.

Other polyadic motifs are less frequent and do not usually form extended stacks like tetrads, but they can significantly alter nucleic acid architecture when present [7]. For example, highly stabilizing motifs, including base triads positioned at the 5’- or 3’-end [8] and in-plane extensions like pentads [9,10], hexads [11], heptads, and octads [12], profoundly influence G4 stability and conformation. Triads in G4s are stabilized by both hydrogen bonding and stacking with end-G-quartets, whereas higher-order polyads, larger than tetrads, though often lacking direct stacking, increase the planar surface area, promoting stacking of entire G4 units. A well-characterized example is the GGA repeat [9,13–17], which generates ultra-stable GNA loops, with the first and last nucleotides forming a sheared G:A base pair, forming a heptad within one plane and integrating the central guanine into another.

Recognizing and understanding the structural levers that govern G4 architecture and folding requires careful identification and analysis not only of the G-quartets themselves but also of additional stabilizing elements, as they can significantly alter the shape and properties of potential binding surfaces for macromolecular partners and small-molecule ligands [4,18–20]. These auxiliary structural elements – such as polyads involving extensions of the G-quartets – also influence how the motif responds to sequence variation; mutations in loop or flanking regions, often considered functionally neutral, can have unexpected effects on folding, stability, and recognition, which is particularly important when designing mutants for structural or functional studies. However, systematic identification of these complex motifs in nucleic acid structures has previously relied on labor-intensive visual inspection, lacking automated and objective solutions.

Here, we present LinkTetrado, a software tool developed to address this gap by enabling robust, automated detection and classification of polyadic motifs within nucleic acid tertiary structures sourced from the Protein Data Bank [21]. LinkTetrado streamlines motif analysis and identification, providing valuable new insights into the landscape and functions of higher-order nucleic acid motifs. The software is freely available at https://github.com/michal-zurkowski/linktetrado.

## Design and implementation

### Automated detection of polyads

**Fig 2:**
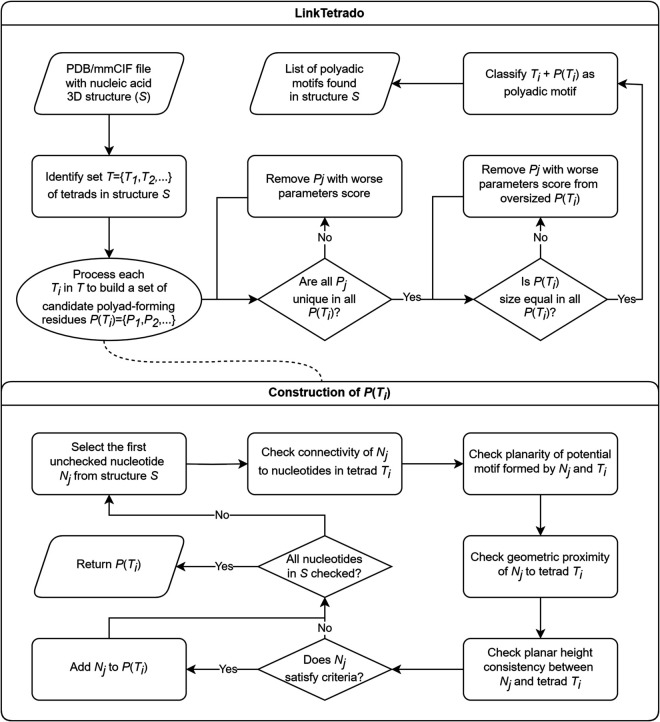
LinkTetrado flowchart.

The proposed algorithm for detecting polyadic motifs operates on three-dimensional structural data provided in PDB or mmCIF format and follows the workflow outlined in Fig 2. It begins by extracting preliminary information about base pairs, stacking interactions, and tetrads using external tools from the RNApolis suite [22]. Based on this input, the algorithm compiles a list of nucleotides that may participate in polyadic motifs. These candidates are subsequently filtered and analyzed in the context of global tetrad stacking to eliminate less probable cases. Finally, all identified polyadic motifs are classified according to the number of participating residues into pentads, hexads, and higher-order structures.

**Fig 3:**
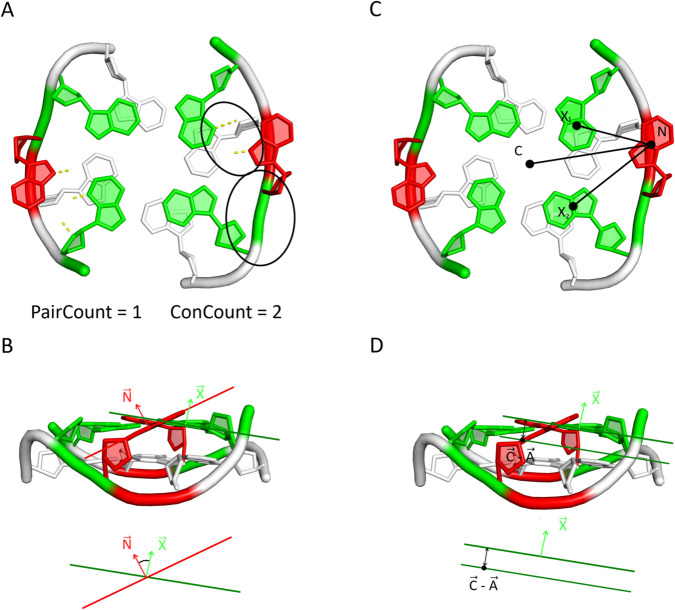
Geometric definitions used in the evaluation of the candidate nucleotide. The schematic illustrates the vectors and reference points employed in (A) Criterion 1 (connectivity), (B) Criterion 2 (tilt, defined by the angle between the candidate and tetrad base normal vectors), (C) Criterion 3 (distance, measured between the candidate and the tetrad centroid), and (D) Criterion 4 (height difference, calculated as the projection of candidate–centroid vectors onto tetrad base normals).

Accurate identification of polyadic motifs involves several steps. First, the algorithm explores the spatial environment of each tetrad *T*_*i*_ in structure *S* to identify all nucleotides that may contribute to the formation of a polyad incorporating *T*_*i*_. The resulting candidates are then subjected to a validation and filtering process based on four structural criteria (Fig 3). These criteria are independent of one another but are evaluated in a predefined order to enable the most effective elimination of unlikely candidates:

Criterion 1: Connectivity to tetrad nucleotides. To check this criterion, the algorithm first evaluates whether the candidate nucleotide *N* is eligible. For this purpose, it computes the connectivity count (*ConCount*), defined as the total number of connections between *N* and the nucleotides of tetrad *T*_*i*_. These connections may arise either from base pairing or from sequential (strand) continuity. A candidate is accepted for further evaluation only if it satisfies the condition ConCount≥2. Once this condition is met, the candidate is scored (Eq 4) using additional parameters, including the number of base pairs it forms with tetrad nucleotides (*PairCount*). By definition, PairCount≤ConCount, since *ConCount* includes both base-pairing and sequential connections, whereas *PairCount* accounts only for base-pairing interactions. This criterion reflects the typical organization of polyadic motifs, including tetrads, where each nucleotide interacts with its two nearest neighbors within the motif.

Criterion 2: Planarity. The algorithm verifies that the candidate nucleotide *N* does not disrupt the overall planarity of the polyadic motif. This is assessed by calculating the tilt of the candidate (*N*) relative to each nucleotide $X_{k}(T_{i})$ (*k*=1..4) in the reference tetrad *T*_*i*_. Tilt is computed as the arccosine of the dot product of the corresponding base normal vectors (Eq 1). Empirical analysis of known tetrads and polyadic motifs shows that the average tilt should not exceed $TT_{AVG}$=40^°^, with an upper limit *TT*_*MAX*_=50^°^ tolerated for individual comparisons. Both, *TT*_*MAX*_ and $TT_{AVG}$ are thresholds used for scoring the candidate (Eq 5); the user can adjust them as desired.

$$Tilt(N,X)=\arccos\left(\vec{N}\cdot \vec{X}\right)$$ (1)

Criterion 3: Geometric proximity to the tetrad. Although base-pair connections (Criterion 1) partially ensure appropriate spatial arrangement, an additional geometric check is applied to confirm that the candidate nucleotide *N* is not positioned too far from tetrad *T*_*i*_. The Euclidean distance is computed between the candidate and the geometric center of the tetrad (i.e., tetrad’s centroid *C*(*T*_*i*_)) using Eq 2, and it must not exceed 13Å. Furthermore, to preserve the approximate symmetry of the polyadic motif, the variation in distances between the candidate and the individual tetrad nucleotides must not exceed 2Å.

$$Dist(N,C)=\sqrt{(N_{x}-C_{x}{)}^{2}+(N_{y}-C_{y}{)}^{2}+(N_{z}-C_{z}{)}^{2}}$$ (2)

Criterion 4: Planar height consistency. Finally, the algorithm verifies whether the candidate nucleotide *N* lies within the approximate plane of tetrad *T*_*i*_. This is done by first identifying the innermost atom $A_{\text{in}}(N)$ of candidate *N* (the atom closest to the tetrad center) and calculating the dot product of the vector $\vec{C}\;-\;\vec{A_{\text{in}}}$, where *C* is the centroid of tetrad *T*_*i*_, with the base normal vector of each nucleotide *X*_*k*_ (*k* = 1..4). The procedure is then repeated for the outermost atom $A_{\text{out}}(N)$ of *N* (the atom involved in the glycosidic bond), using the vector $\vec{C}\;-\;\vec{A_{\text{out}}}$. For both atoms, the resulting height difference relative to the tetrad plane must not exceed *HT*_*MAX*_=3.7Å for any *X*_*k*_ (Eq 3), and the average for all *X*_*k*_ cannot exceed $HT_{AVG}$=3.15Å. This criterion is relatively permissive and may occasionally allow a nucleotide to be considered as a candidate for multiple tetrads; however, such cases are resolved during the final filtering stage. Both, *HT*_*MAX*_ and $HT_{AVG}$ are used to score the candidate (Eq 5); the user can adjust them as desired.

$$\Delta H(C,A,X)=(\vec{C}-\vec{A})\cdot \vec{X}$$ (3)

The final stage of polyad detection involves refining the pool of candidate partners for each tetrad through a two-step process: (1) ensuring that no nucleotide is assigned as a partner for multiple tetrads, and (2) standardizing the order of all detected polyadic motifs within a stacked tetrad formation. Each candidate—the T tetrad and the extending nucleotide N—identified by the algorithm is assigned a score based on Criteria 1–4 (Eq 4), reflecting how well it satisfies the defined cutoff thresholds. If a nucleotide is eligible to participate in polyadic motifs with more than one tetrad, the scoring function is used to assign it to the most likely tetrad. Once the final candidate list is established for each tetrad, the algorithm adjusts motif compositions to ensure that all polyadic motifs within the stacked formation have the same order. In cases of discrepancy, higher-order motifs are reduced by removing the lowest-scoring candidates while preserving the most likely ones. This reduction also considers the spatial arrangement of nucleotides to maintain the overall symmetry of the polyadic structure.

$$Score(N,T)=\frac{\sum_{i=1}^{6}S_{i}(N,T)}{2+PairCount}$$ (4)

where:

$$S_{1}(N,T)=1.0\;*\;((TT_{AVG}-|Avg(Tilt(N,X_{1})...Tilt(N,X_{4}))|)\;*\;100{)}^{2}\;S_{2}(N,T)=0.5\;*\;((TT_{MAX}-|Max(Tilt(N,X_{1})...Tilt(N,X_{4}))|)\;*\;100{)}^{2}\;S_{3}(N,T)=1.0\;*\;((HT_{AVG}-|Avg(\Delta H(C,A_{in},X_{1})...\Delta H(C,A_{in},X_{4}))|)\;*\;100{)}^{1.75}\;S_{4}(N,T)=0.75\;*\;((HT_{MAX}-|Max(\Delta H(C,A_{in},X_{1})...\Delta H(C,A_{in},X_{4}))|)\;*\;100{)}^{1.75}\;S_{5}(N,T)=1.0\;*\;((HT_{AVG}-|Avg(\Delta H(C,A_{out},X_{1})...\Delta H(C,A_{out},X_{4}))|)\;*\;100{)}^{1.75}\;S_{6}(N,T)=0.75\;*\;((HT_{MAX}-|Max(\Delta H(C,A_{out},X_{1})...\Delta H(C,A_{out},X_{4}))|)\;*\;100{)}^{1.75}$$ (5)

$TT_{\text{avg}}$, $TT_{\text{max}}$, $HT_{\text{avg}}$, and $HT_{\text{max}}$ denote the thresholds for the average and maximum values of tilt and height differences, respectively. The function *Avg*() returns the mean of the four tilt values or four height differences supplied as arguments, while *Max*() returns the largest value among its arguments.

### LinkTetrado tool

LinkTetrado is a Python 3 command-line application designed to process tertiary structure data of nucleic acid molecules. It relies on standard Python libraries and two additional packages from the RNApolis suite, both available via the PyPI repository: RNApolis Annotator, which identifies base pairs and stacking interactions in 3D structures, and ElTetrado, which performs initial tetrad detection [22,23]. The tool is compatible with all major operating systems and can be downloaded from the GitHub repository (https://github.com/michal-zurkowski/linktetrado) or installed directly from PyPI.

To run the program, users invoke it via the command line by specifying its name along with the --input parameter, followed by the input structure file in PDB or mmCIF format. The program automatically recognizes gzip-compressed files and processes them without requiring manual decompression. For advanced functionality, users can provide passthrough parameters to customize the ElTetrado analysis. One such optional parameter is --lax-order, which adjusts the algorithm’s filtering mode, enabling the detection of polyads with varying orders within a single stack. If this option is not enabled, all polyads are classified at the highest common order to ensure consistent representation within the stacking.

Additionally, the program provides parameters that allow users to adjust candidate scoring. These include --height-max and --height-avg, which define the maximum and average height thresholds for evaluating whether a nucleotide lies within the plane of a tetrad, and --tilt-max and --tilt-avg, which set the corresponding thresholds for candidate-tetrad plane consistency. Furthermore, --dist-in-max and --dist-out-max control the search range for candidate nucleotides located farther from the tetrad center, based on their innermost and outermost atoms, respectively. By tuning these parameters, users can adjust the stringency of candidate selection to match their specific requirements.

The output includes detailed information on the polyadic motifs identified in the input structure: (i) a list of detected polyadic motifs with nucleotide-level details and final classifications, and (ii) a list of tetrads contributing to stacking, located either between or at the ends of polyads.

## Results

### Polyads found in PDB structures

LinkTetrado was run on a dataset of 529 nucleic acid structures containing G-quadruplexes, retrieved from the ONQUADRO database (accessed in February 2024) [24]. These structures represent all entries currently deposited in the Protein Data Bank (PDB) that contain quadruplexes and consist of at least two tetrads. Of these, 25 structures were found to contain polyadic motifs. Six of the 25 structures appeared multiple times in the dataset due to alternative assemblies deposited under the same PDB ID. To avoid redundancy, only one representative assembly per structure was retained for further analysis. Among the 25 non-redundant structures with polyads, 15 (60%) were RNA, 9 (36%) were DNA, and 1 (4%) was a hybrid RNA/DNA structure. In terms of experimental methods, the majority were determined using NMR (Nuclear Magnetic Resonance) spectroscopy (14 structures, 56%), while the remaining 11 (44%) were solved via X-ray crystallography.

The algorithm successfully identified 40 polyadic motifs interacting with a total of 35 quadruplexes across the 25 non-redundant structures (see S1 Table). Of these, 26 quadruplexes (74%) were canonical G-quadruplexes, while the remaining 9 (26%) included tetrads formed by a mixture of nucleotides, although most still contained G-tetrads. Importantly, polyadic motifs in all but two cases were formed exclusively with G-tetrads. The remaining two polyads were based on A-tetrads, representing rare instances of non-G-based higher-order interactions.

**Fig 4:**
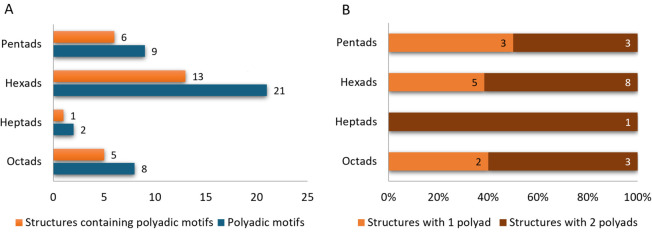
(A) The number of structures containing polyadic motifs, grouped by motif type, and the total number of motifs of each type detected by LinkTetrado. (B) Distribution of structures containing only one or two polyadic motifs of a given type. Each bar shows both the percentage and the absolute number of structures.

LinkTetrado correctly identified 9 pentads, 21 hexads, 2 heptads, and 8 octads. Eight of these polyads were explicitly described in the associated publications. The frequency and structural distribution of the polyadic motifs are summarized in Fig 4, which also indicates the number of distinct structures containing each motif type. Notably, pentads appeared in 6 structures, hexads in 13, heptads in only 1, and octads in 5. This distribution directly reflects the variation in the number of motifs per structure. While 40% of the structures featured a single polyadic motif, 60% contained multiple occurrences. For example, among the 13 structures with hexads, 8 (62%) contained two hexads, while 5 contained one. A similar pattern was observed for octads and pentads. Both heptads occurred in the same structure.

In most identified polyads, guanine was the only base type forming canonical tetrads. Additional nucleotides that extended these tetrads into polyads included adenines (61%), uridines (33%), and thymines (6%). These percentages refer to the identities of residues directly interacting with the previously identified tetrads. Notably, no cytosines or additional guanines outside the tetrads were involved in the formation of polyads.

**Fig 5:**
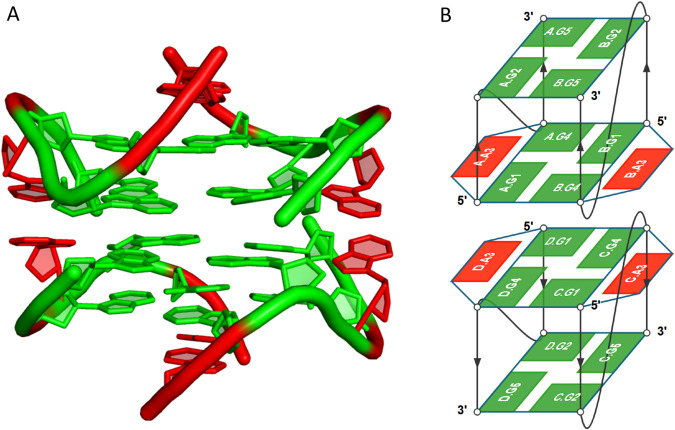
Dimeric DNA structure (PDB ID: 1EEG; [25]) shown as (A) a cartoon model visualized in PyMOL [26] and (B) a layer diagram generated with DrawTetrado [27]. The G4 helix is colored green and contains two (A:)G:G:G:G(:A) hexads. Adenines that interact with tetrad-forming guanines are highlighted in red.

We observed that motifs with an even number of nucleotides – such as tetrads, hexads, and octads – tend to maintain symmetrical arrangements, which likely contribute to increased structural stability. Hexads were detected more frequently than pentads, possibly due to the added structural rigidity provided by their symmetrical configuration. Although octads share similar symmetry-related advantages, their higher structural complexity may explain their lower frequency of occurrence. Notably, all identified octads were found in RNA or RNA/DNA hybrid structures and were exclusively determined by X-ray crystallography. Furthermore, we identified helical arrangements containing two quadruplexes, in which the terminal ends were capped by multimeric motifs (Fig 5).

LinkTetrado also produced six false positives – incorrectly identified pentads. These cases were identified through manual validation based on NMR restraints and visual inspection and were excluded from the results presented above.

### Validation with experimental data

The majority of structures deposited in the Protein Data Bank have been determined using X-ray crystallography, cryo-electron microscopy (cryo-EM), or Nuclear Magnetic Resonance spectroscopy (NMR). Each of these techniques provides structural information through fundamentally different approaches. While X-ray and cryo-EM offer relatively direct visualizations of structural features such as polyads, NMR infers these motifs more indirectly – primarily through analysis of nuclear Overhauser effect (NOE) contacts and stable hydrogen bonds. Given these methodological differences, we found it essential to validate the polyads identified by LinkTetrado against the original experimental data, particularly for NMR-derived structures, as molecular simulations used to generate structural models from NMR data can occasionally yield polyads not fully supported by experimental restraints. LinkTetrado supports such verification by allowing users to assess whether the predicted polyadic motif is consistent with the available data. However, the validation process itself requires careful manual inspection of NMR restraint files, chemical shift assignment, and related publications.

To ensure the reliability of our results, all polyadic motifs identified by LinkTetrado were manually validated against experimental data. Based on this verification, we found that the algorithm identified 40 true positives (TP) and 6 false positives (FP). Thus, its precision (also known as positive predictive value), calculated according to Eq 6, is 0.87. Precision is defined as the proportion of true positives among all instances predicted as positive (i.e., true positives + false positives). In this case, a precision of 0.87 indicates that 87% of the motifs identified by the algorithm are indeed correct, which is a very good result, especially given the complexity of detecting polyadic interactions.

$$Precision=\frac{TP}{TP+FP}$$ (6)

Below, we present a representative true positive case in which two identified motifs were supported by structural restraints derived from NMR spectroscopy. The example involves the solution structure of a potassium-stabilized dimeric RNA quadruplex containing two G(:A):G:G(:A):G hexads, two canonical G:G:G:G tetrads, and 3’ stacked adenine residues (PDB ID: 2RQJ) [16]. LinkTetrado correctly identified both G(:A):G:G(:A):G hexads, which extend the canonical G-tetrad framework through additional interactions. The first hexad consists of residues G1, A3, G4, G7, A9, and G10 from chain A. Among these, G1, G4, G7, and G10 form a planar tetrad, while A3 and A9 extend this core into a higher-order polyad. The second hexad comprises residues G13, A15, G16, G19, A21, and G22 from chain B, with G13, G16, G19, and G22 forming the tetrad core, and A15 and A21 acting as extensions. In both cases, the hexads represent extended G-tetrad systems stabilized by sheared G:A base pairs.

**Fig 6:**
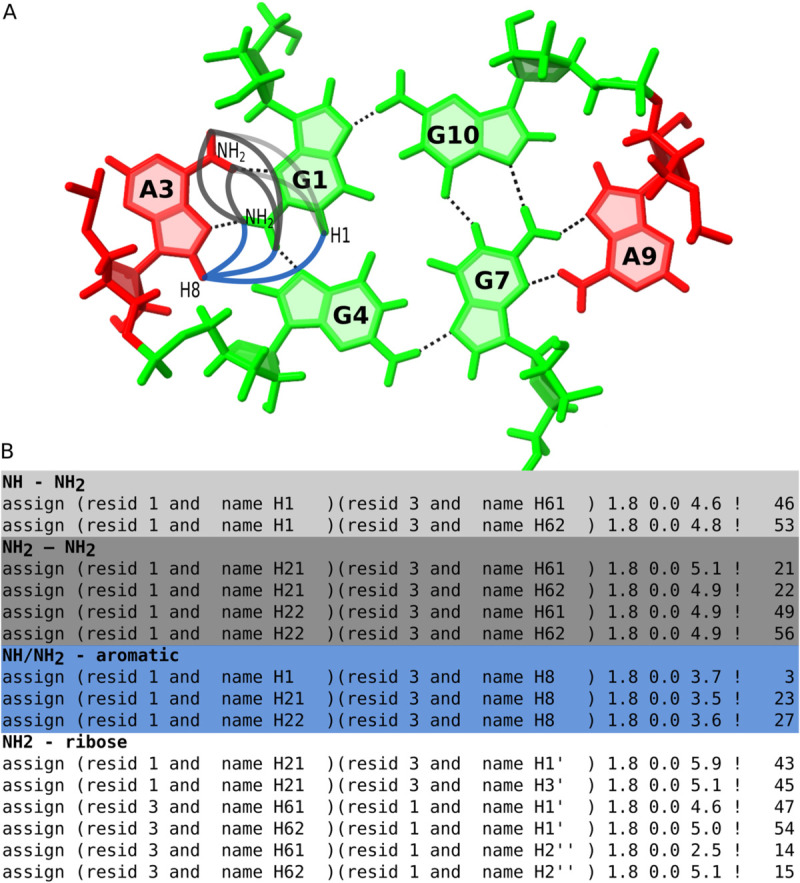
Confirmation of polyad formation using NMR restraints for the dimeric RNA G-quadruplex structure (PDB ID: 2RQJ). (A) Schematic representation of distance restraints between exchangeable protons and their partners in residues G1 and A3, which form a sheared G:A base pair underlying hexad formation. (B) Selected lines from the 2rqj.mr restraint file that report on the in-plane position of residue A3 and the formation of hydrogen bonds between G1 and A3. For clarity, only restraints involving exchangeable protons are shown. The lines in (B) are shaded in colors that correspond to the interaction paths marked in (A), indicating which restraint supports which structural feature.

NMR restraint data deposited in the Protein Data Bank for this structure allow us to cross-check experimental NOE-based evidence supporting the existence of hexads. The reported NOE distance restraints confirm specific interatomic contacts between guanine and adjacent adenine residues, thereby validating the proposed base-pairing patterns. In particular, interactions between G1 and A3, and between G7 and A9 in the first hexad (Fig 6), as well as between G13 and A15, and between G19 and A21 in the second hexad, are supported by NOEs involving G-NH2 and A-H8, G-H1 and A-H8, and G-H1 and A-NH2 protons. Furthermore, additional restraints involving the amino groups of guanine and adenine residues, as well as multiple NOE contacts between guanine sugar protons (H1’, H4’, H5’) and adenine amino protons, and *vice versa* (Fig 6B), further support the specific interactions required for hexad formation. Additional evidence comes from chemical shift data [16], which show that both protons of the amino groups of G1, G7, G13, and G19 are downfield-shifted. This is consistent with their involvement in hydrogen bonding. Meanwhile, the observable signals of the amino groups of A3, A9, A15, and A21 reinforce their role in hexad stabilization. Taken together, these observations confirm the presence of extended base-pairing networks and highlight the stabilizing role of adenine residues in the G-rich hexads identified in the analyzed structure.

**Fig 7:**
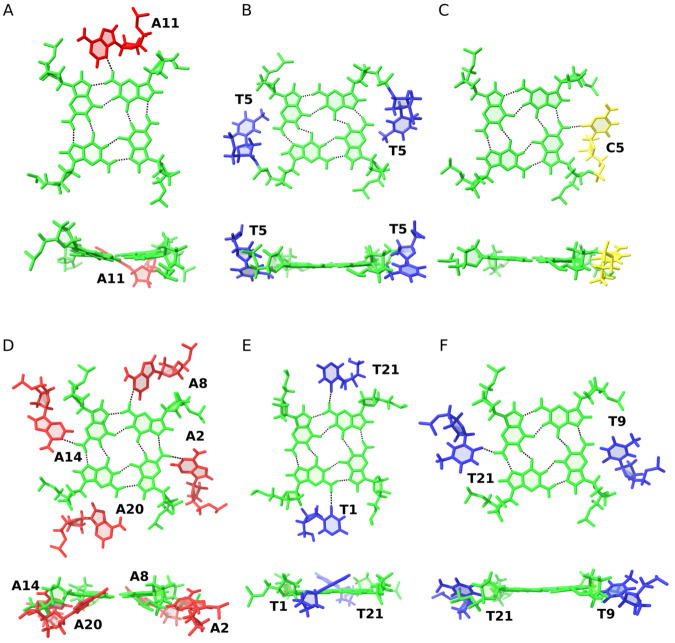
False positives identified by LinkTetrado in structures with PDB IDs (A) 5LIG, (B) 1NYD, (C) 2KYP, (D) 6H5R, (E) 4R44, and (F) 6W9P. Top and side views of each structure illustrate the position of the hypothetical polyad residue relative to the G-quartet plane. Hydrogen bonds detected using molecular visualization software are shown in black. Residues are color-coded: green for guanine, red for adenine, blue for thymine, and yellow for cytosine.

In contrast to the validated example described above, we also encountered cases where the available experimental evidence did not support polyadic motifs identified by LinkTetrado. One such false positive was found in the structure of the G-quadruplex formed at the 5’-end of NHEIII_1 element in human c-MYC promoter (PDB ID: 5LIG), where the algorithm identified a pentad formed by A11, G5, G9, G14, and G18 (Fig 7A). A careful examination of the NMR restraint files and accompanying publication [28] revealed no NOE contacts or chemical shift data consistent with the proposed interactions. Notably, both the NMR restraints and chemical shift data for this structure are deposited in the Protein Data Bank, allowing for a quick verification. In particular, the NMR restraints do not indicate any specific conformation of A11. Visual inspection of the structure confirmed that A11 is brought into proximity with the G5:G9:G14:G18 quartet by a two-residue propeller loop. However, the Hoogsteen edge of A11 is not oriented toward any potential pairing partner within the G-quartet. Instead, A11 approaches the quartet via its sugar edge, which is rarely involved in the formation of pentads in G-quadruplexes.

A further false positive, arising from the absence of decisive restraints or observations supporting polyad formation, was identified in the tetrameric G-quadruplex formed by the GCGGTGGAT repeat (PDB ID: 1NYD; [29]). This structure, which assembles as a dimer of dimers, exhibits unique features such as a GC tetrad and AA mismatches. LinkTetrado found a hexad, consisting of a canonical G-quartet (G3:G6:G3:G6) flanked by two thymine residues (T5) contributed by one-nucleotide propeller loops (Fig 7B). According to the NMR restraint file, the positions of the two T5 residues are defined by restraints involving their methyl and sugar protons from adjacent nucleotides (C2, G3, G4), as well as sugar protons of T5 and sugar protons of G4, and sequential contacts between T5 H1’/3’ and G6 H8. The imino proton of T5 does give rise to a detectable resonance; however, its hydrogen-bonding partner was not identified in the original publication [29]. Moreover, NOE contacts suggest that the Watson-Crick edge of T5 points away from the G-quadruplex core. Despite T5 exhibiting an unusually high number of contacts for a residue located in a single-nucleotide propeller loop, its geometry is incompatible with planar hexad formation. Structural analysis shows that T5 is not coplanar with the G-quartet and that its hydrogen-bond donor and acceptor groups are directed away from the quartet, thereby precluding formation of the hydrogen-bonding network required for a stable polyad.

The third false positive was identified in the structure involving a G-quadruplex derived from the c-kit promoter (PDB ID: 2KYP; [30]), which adopts a parallel three-quartet conformation. LinkTetrado detected a pentad comprising the G-quartet (G4:G8:G16:G20) and C5 from a one-nucleotide propeller loop (Fig 7C). NMR restraints reveal correlations between G4 H4’ and C5 H1’, and importantly between C5 H2’/” and C9 H5, the latter being the first residue of an adjacent propeller loop. This interaction was highlighted by the authors as a key cross-talk mechanism between loops, with C5 stacking on C9 contributing to structural stabilization [30]. In this conformation, C5 is positioned near G4, and structures deposited in the Protein Data Bank suggest a potential hydrogen bond between the C5 O2 atom and G4 H22. However, the available literature does not conclusively confirm the presence of a stable G4 H22 resonance [30]. Given the nature of the atoms involved and the inherent flexibility of the loop, such a hydrogen bond would likely be challenging to detect by NMR. Consequently, the observed pentad most likely results from a specific loop conformation stabilized by stacking interactions rather than stable polyad formation involving direct hydrogen bonding between C5 and the G-quartet.

Another three false positives were found in X-ray structures. The first one (Fig 7D) represents a complex between a human telomeric sequence and a gold(I) dicarbene anticancer drug (PDB ID: 6H5R; [31]). The adenines identified by Linktetrado as part of an octad originate from TTA propeller loops. These adenine residues are oriented toward the groove, and although molecular visualization software detects hydrogen bonds between the N1 atom of adenines and the NH group of guanines in the G-quartets, their out-of-plane conformation weakens such interactions. The original study focused on the binding mode of the drug within the complex and did not recognize the presence of a polyad.

The example shown in Fig 7E, where LinkTetrado detected a hexad, corresponds to crystals formed from a racemic mixture of L- and D-DNA (PDB ID: 4R44; [32]). While hydrogen bonds can be observed between the O2 atom of thymine and the NH groups of guanines in the G-quartets, these interactions are absent in the second subunit of the crystal. The displacement of the thymine residue from the G-quartet plane suggests that, if such hydrogen bonds occur, they are weak and unlikely to contribute significantly to hexad stabilization.

The final false positive (Fig 7F) involves a hexad identified by Linktetrado in the structure with PDB ID 6W9P, a variant of the *T. thermophila* telomeric sequence [33] forming a parallel G-quadruplex with four G-quartets. The two thymine residues identified as part of the hexad, T9 and T21, have the highest B-factors among all loop thymine residues, indicating significant flexibility. While other loop residues are stabilized through interactions with neighboring subunits, the elevated B-factors of T9 and T21 suggest that they are not involved in stabilizing contacts and do not contribute meaningfully to hexad formation.

## Availability and future directions

This work presents LinkTetrado, the first fully automated method for detecting polyadic motifs in the three-dimensional structures of nucleic acids. The software is freely available at https://github.com/michal-zurkowski/linktetrado, and the structures analyzed in this study can be accessed through the ONQUADRO database at https://onquadro.cs.put.poznan.pl.

Future developments of LinkTetrado will focus on improving both detection accuracy and user control. A key enhancement will involve the implementation of adjustable detection thresholds, enabling users to fine-tune the sensitivity and specificity of motif recognition depending on the context of analysis. This added flexibility will allow the tool to be adapted for different use cases, from exploratory analyses to more stringent structural annotation. Another planned advancement is the integration of support for experimental NMR data, including restraints and chemical shift information. This would allow users to incorporate experimental evidence directly into the detection pipeline, improving the biological relevance and reliability of the identified motifs. In the longer term, we would like to incorporate a machine learning-based detection module, once a sufficiently large and diverse dataset of validated polyadic motifs becomes available. Such a model could help capture subtle geometric and chemical features that are difficult to encode through rule-based approaches, offering a more generalized and data-driven extension of the current algorithm. While this goal remains aspirational at present, it outlines a promising direction for future research and development.

## Supporting information

S1 Table PDB structures containing polyads identified by LinkTetrado. (XLSX)
